# Supplementary material for: Structure and functions of Yellow-breasted Boubou (Laniarius atroflavus) solos and duets
Source: PeerJ. 2020 Oct 21;8:e10214. doi: 10.7717/peerj.10214 (PMC7585374; doi:10.7717/peerj.10214)
Supplement: Supplemental Information 1 — Each row from 1 to 46 indicate different classes of recorded bouts. Rows in the column ‘Song or call bout’ letters indicate all combinations of vocalizations produced by the study species. E.g., ‘H’ in row 1 indicates that High whee-oo solos produced by males (without response from a female) were recorded 1697 times, which gives 28,6% of all bouts etc. The next row 2 indicates that we recorded 354 ‘HK’ cases in which High whee-oo male song phrase was answered by Kee-roo phrase of a female, and this gives 6% all bouts, or 8.8% among bouts initiated by males etc.Colours left for easier recognition between male (yellow) and female (green) initiated vocalizations. Abbreviations of song and call types: males: H –High whee-oo, L –Low whee-oo, W –Hwee-hwee; females: C –Chock, Cs –Chock-series, K –Kee-roo, Q –Keck, R –Rasp. [file peerj-08-10214-s001.docx]

| No | Song or call bout | Frequency | Initiator sex | Initiator call type | Answered | % all bouts | % bouts within a male or female bout initiator | % within a sex/call type | % within sex/call answered | % within solos | % within male or female solos | % within duets | % within male or female initiated duets |
| --- | --- | --- | --- | --- | --- | --- | --- | --- | --- | --- | --- | --- | --- |
| 1 | H | 1697 | Male | H | no | 28,6% | 42,3% | 73,3% |  | 37,9% | 60,0% |  |  |
| 2 | HK | 354 | Male | H | yes | 6,0% | 8,8% | 15,3% | 57,3% |  |  | 24,2% | 29,9% |
| 3 | HC | 257 | Male | H | yes | 4,3% | 6,4% | 11,1% | 41,6% |  |  | 17,6% | 21,7% |
| 4 | HR | 3 | Male | H | yes | 0,1% | 0,1% | 0,1% | 0,5% |  |  | 0,2% | 0,3% |
| 5 | HCs | 2 | Male | H | yes | 0,0% | 0,0% | 0,1% | 0,3% |  |  | 0,1% | 0,2% |
| 6 | HQ | 1 | Male | H | yes | 0,0% | 0,0% | 0,0% | 0,2% |  |  | 0,1% | 0,1% |
| 7 | HLC | 1 | Male | H | yes | 0,0% | 0,0% | 0,0% | 0,2% |  |  | 0,1% | 0,1% |
| 8 | L | 709 | Male | L | no | 11,9% | 17,7% | 60,8% |  | 15,9% | 25,1% |  |  |
| 9 | LK | 326 | Male | L | yes | 5,5% | 8,1% | 27,9% | 71,2% |  |  | 22,3% | 27,5% |
| 10 | LC | 126 | Male | L | yes | 2,1% | 3,1% | 10,8% | 27,5% |  |  | 8,6% | 10,6% |
| 11 | LR | 5 | Male | L | yes | 0,1% | 0,1% | 0,4% | 1,1% |  |  | 0,3% | 0,4% |
| 12 | LHK | 1 | Male | L | yes | 0,0% | 0,0% | 0,1% | 0,2% |  |  | 0,1% | 0,1% |
| 13 | W | 421 | Male | W | no | 7,1% | 10,5% | 79,4% |  | 9,4% | 14,9% |  |  |
| 14 | WK | 61 | Male | W | yes | 1,0% | 1,5% | 11,5% | 56,0% |  |  | 4,2% | 5,1% |
| 15 | WC | 45 | Male | W | yes | 0,8% | 1,1% | 8,5% | 41,3% |  |  | 3,1% | 3,8% |
| 16 | WQ | 1 | Male | W | yes | 0,0% | 0,0% | 0,2% | 0,9% |  |  | 0,1% | 0,1% |
| 17 | WLK | 1 | Male | W | yes | 0,0% | 0,0% | 0,2% | 0,9% |  |  | 0,1% | 0,1% |
| 18 | WHC | 1 | Male | W | yes | 0,0% | 0,0% | 0,2% | 0,9% |  |  | 0,1% | 0,1% |
| 19 | Cs | 534 | Female | Cs | no | 9,0% | 27,8% | 81,9% |  | 11,9% | 32,5% |  |  |
| 20 | CsHK | 76 | Female | Cs | yes | 1,3% | 4,0% | 11,7% | 64,4% |  |  | 5,2% | 27,4% |
| 21 | CsLK | 23 | Female | Cs | yes | 0,4% | 1,2% | 3,5% | 19,5% |  |  | 1,6% | 8,3% |
| 22 | CsH | 12 | Female | Cs | yes | 0,2% | 0,6% | 1,8% | 10,2% |  |  | 0,8% | 4,3% |
| 23 | CsWK | 3 | Female | Cs | yes | 0,1% | 0,2% | 0,5% | 2,5% |  |  | 0,2% | 1,1% |
| 24 | CsWLK | 2 | Female | Cs | yes | 0,0% | 0,1% | 0,3% | 1,7% |  |  | 0,1% | 0,7% |
| 25 | CsL | 1 | Female | Cs | yes | 0,0% | 0,1% | 0,2% | 0,8% |  |  | 0,1% | 0,4% |
| 26 | CsHLK | 1 | Female | Cs | yes | 0,0% | 0,1% | 0,2% | 0,8% |  |  | 0,1% | 0,4% |
| 27 | K | 57 | Female | K | no | 1,0% | 3,0% | 37,0% |  | 1,3% | 3,5% |  |  |
| 28 | KL | 54 | Female | K | yes | 0,9% | 2,8% | 35,1% | 55,7% |  |  | 3,7% | 19,5% |
| 29 | KW | 22 | Female | K | yes | 0,4% | 1,1% | 14,3% | 22,7% |  |  | 1,5% | 7,9% |
| 30 | KH | 17 | Female | K | yes | 0,3% | 0,9% | 11,0% | 17,5% |  |  | 1,2% | 6,1% |
| 31 | KHL | 1 | Female | K | yes | 0,0% | 0,1% | 0,6% | 1,0% |  |  | 0,1% | 0,4% |
| 32 | KHW | 1 | Female | K | yes | 0,0% | 0,1% | 0,6% | 1,0% |  |  | 0,1% | 0,4% |
| 33 | KLW | 1 | Female | K | yes | 0,0% | 0,1% | 0,6% | 1,0% |  |  | 0,1% | 0,4% |
| 34 | KWHL | 1 | Female | K | yes | 0,0% | 0,1% | 0,6% | 1,0% |  |  | 0,1% | 0,4% |
| 35 | C | 63 | Female | C | no | 1,1% | 3,3% | 53,8% |  | 1,4% | 3,8% |  |  |
| 36 | CL | 22 | Female | C | yes | 0,4% | 1,1% | 18,8% | 34,4% |  |  | 1,5% | 7,9% |
| 37 | CH | 21 | Female | C | yes | 0,4% | 1,1% | 17,9% | 32,8% |  |  | 1,4% | 7,6% |
| 38 | CW | 10 | Female | C | yes | 0,2% | 0,5% | 8,5% | 15,6% |  |  | 0,7% | 3,6% |
| 39 | CHWL | 1 | Female | C | yes | 0,0% | 0,1% | 0,9% | 1,6% |  |  | 0,1% | 0,4% |
| 40 | Q | 972 | Female | Q | no | 16,4% | 50,6% | 99,8% |  | 21,7% | 59,1% |  |  |
| 41 | QL | 1 | Female | Q | yes | 0,0% | 0,1% | 0,1% | 0,1% |  |  | 0,1% | 0,4% |
| 42 | QW | 1 | Female | Q | yes | 0,0% | 0,1% | 0,1% | 0,1% |  |  | 0,1% | 0,4% |
| 43 | R | 19 | Female | R | no | 0,3% | 1,0% | 76,0% |  | 0,4% | 1,2% |  |  |
| 44 | RL | 4 | Female | R | yes | 0,1% | 0,2% | 16,0% | 66,7% |  |  | 0,3% | 1,4% |
| 45 | RW | 1 | Female | R | yes | 0,0% | 0,1% | 4,0% | 16,7% |  |  | 0,1% | 0,4% |
| 46 | RWL | 1 | Female | R | yes | 0,0% | 0,1% | 4,0% | 16,7% |  |  | 0,1% | 0,4% |
